# Targeting the Calcium Signalling Machinery in Cancer

**DOI:** 10.3390/cancers12092351

**Published:** 2020-08-20

**Authors:** Jason I. E. Bruce, Andrew D. James

**Affiliations:** 1 Division of Cancer Sciences, School of Medical Sciences, Faculty of Biology, Medicine and Health, University of Manchester, Manchester M13 9PT, UK; 2 Department of Biology, University of York, Heslington, York YO10 5DD, UK; andrew.james@york.ac.uk

**Keywords:** calcium signalling, cancer, cancer hallmarks, TRP channels, CRAC, Orai, STIM, PMCA, SERCA, SPCA, IP3R, cell death, cell proliferation, cell migration, invasion, metastasis

## Abstract

Cancer is caused by excessive cell proliferation and a propensity to avoid cell death, while the spread of cancer is facilitated by enhanced cellular migration, invasion, and vascularization. Cytosolic Ca^2+^ is central to each of these important processes, yet to date, there are no cancer drugs currently being used clinically, and very few undergoing clinical trials, that target the Ca^2+^ signalling machinery. The aim of this review is to highlight some of the emerging evidence that targeting key components of the Ca^2+^ signalling machinery represents a novel and relatively untapped therapeutic strategy for the treatment of cancer.

## 1. Introduction

**Figure 1:**
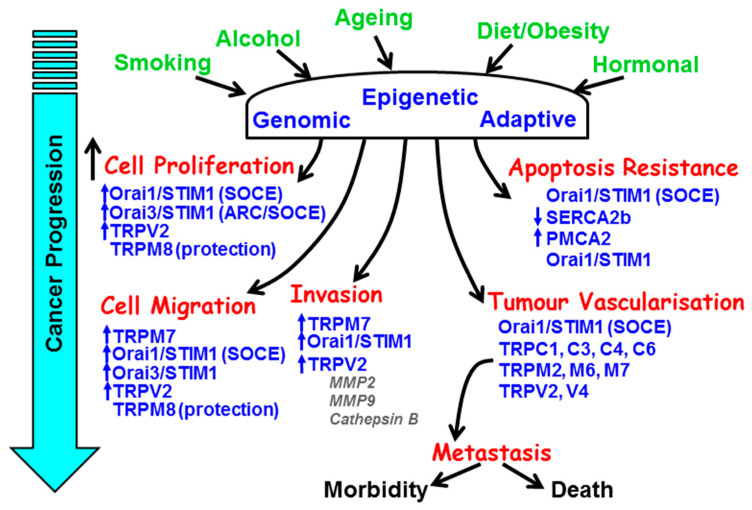
The remodelling of key components of the Ca^2+^ signalling machinery responsible for the hallmark processes of cancer during cancer progression. The major causes and risk factors of cancer induce genomic, epigenetic, and adaptive changes in key Ca^2+^ transporters responsible for the increase in cell proliferation, resistance to apoptosis, cellular migration and invasion, and tumour vascularisation.

The major hallmarks of the cancer phenotype include limitless replicative capacity, self-sufficiency of growth and/or insensitivity of anti-growth signals, resistance to apoptosis, tissue invasion, and angiogenesis [1]. The vast majority of mainstay chemotherapeutic anti-cancer drugs target DNA replication (etoposide/gemcitibine), DNA/RNA synthesis (paclitaxel), DNA damage (doxorubicin), and growth factor receptor (GFR) signalling (Iressa, erlotinib) [2]. These all have the desired effects of stopping cells from dividing or triggering cell death. However, cytosolic Ca^2+^ and the components of the Ca^2+^ signalling machinery have critical roles in the regulation of all the above hallmark processes that underlie the cancer phenotype (Figure 1).

It is thus surprising that none of the current mainstay cancer chemotherapy drugs target the Ca^2+^ signalling machinery. This may well change in the future, especially since the recent discovery of the molecular identity of novel Ca^2+^ channels (e.g., Orai1 and numerous members of the transient receptor protein (TRP) family) and key regulatory proteins (e.g., stromal interacting molecule (STIM) and secretory pathway ATPase (SPCA)). Emerging evidence shows that these proteins have an increasingly important role in the numerous hallmark processes underlying the cancer phenotype, and thus offer a rich tapestry of novel putative therapeutic targets. Moreover, the cell surface expression of some of these Ca^2+^ channels and transporters makes them highly accessible to novel drugs or even antibody therapy. However, it is also important to note that the versatility and ubiquitous nature of Ca^2+^ signalling proteins means that targeting them with novel therapeutics would likely produce unacceptable adverse effects regardless of their specificity. Therefore, the ideal strategy is to target Ca^2+^ channels, or regulatory proteins, that are either uniquely expressed or their expression results in an entirely new function in cancer cells. This review will thus focus on candidates that fulfil some of these important criteria.

## 2. The Versatility of Ca^2+^ Signalling

Cytosolic calcium (Ca^2+^) is arguably the most important cell signal in biology, controlling diverse cellular process from the very beginning of cell life through to cell death and almost everything in between. This is achieved because Ca^2+^ signals come in different shapes, both in time and space, and are regulated by a diverse repertoire of Ca^2+^ channels, transporters, pumps (ATPases), and binding proteins, collectively known as the Ca^2+^ signalling machinery [3]. Key components of the Ca^2+^ signalling machinery work in concert to generate complex spatiotemporal patterns of Ca^2+^ signalling, such as Ca^2+^ oscillations, Ca^2+^ waves, spatially restricted Ca^2+^ spikes, and sustained global Ca^2+^ signals. These different spatiotemporal patterns of Ca^2+^ signalling can differentially encode diverse physiological processes, from cell division, transcription and differentiation, cellular contractility and motility, to fluid and protein secretion, and ultimately cell death [3]. Importantly, different types of Ca^2+^ signal can activate diametrically opposed cellular responses within the same cell. For example, global Ca^2+^ waves control the contraction of smooth muscle cells, whereas localised Ca^2+^ release specifically activates Ca^2+^-dependent K^+^ channels, hyperpolarisation, and the consequent smooth muscle relaxation [4]. Moreover, the temporal properties of Ca^2+^ signalling convey important information. For example, the frequency of [Ca^2+^]_i_ oscillations can differentially regulate gene transcription, and thus cell fate decisions, in T lymphocytes [5,6].

## 3. The Calcium Signalling Machinery

The Ca^2+^ signalling machinery can be broadly categorised into those that elevate cytosolic Ca^2+^ (such as Ca^2+^ entry channels and intracellular Ca^2+^ release channels) and those that reduce cytosolic Ca^2+^ (such as Ca^2+^ pumps, Ca^2+^ transporters, and Ca^2+^-binding proteins). Intracellular Ca^2+^ release channels primarily include inositol 1,4,5-trisphosphate (IP_3_)-gated Ca^2+^ channels (also known as IP_3_ receptors (IP_3_Rs)) and ryanodine receptors (RyRs), both expressed on the endoplasmic reticulum or sarcoplasmic reticulum. Both IP_3_Rs and RyRs can be gated by Ca^2+^ and contribute to Ca^2+^ induced Ca^2+^ release (CICR), which is important for amplifying Ca^2+^ signals [7,8]. Moreover, IP_3_Rs are inhibited by high Ca^2+^ concentration and represent the intrinsic oscillatory mechanism in many non-excitable cells [7,8]. Additional Ca^2+^ release channels have been shown to be expressed on other organelles such as lysosomes and secretory vesicles, gated by nicotinic acid adenine dinucleotide (NAADP) [9].

Ca^2+^ entry channels can be categorised into voltage-operated Ca^2+^ channels (VOCCs) activated by changes in membrane potential (e.g., L, T, N, and P/Q-type Ca^2+^ channels), ligand-gated Ca^2+^ channels (e.g., glutamate-gated N-Methyl D-glutamate (NMDA) receptors, ATP-gated purinergic P2X receptors), store operated Ca^2+^ entry channels (SOCE), and transient receptor potential (TRP) family of ion channels. Store-operated Ca^2+^ entry (SOCE) channels, otherwise known as Ca^2+^ release-activated Ca^2+^ channels (CRAC) or capacitative Ca^2+^ entry (CCE), are activated following depletion of the ER/SR Ca^2+^ store and have been studied extensively since they were first described by Jim Putney during the early 1980s [10]. However, the molecular mechanism underlying SOCE remained elusive for over 20 years until the discovery of two important proteins, stromal interacting molecule (STIM) and CRACM (CRAC modulators), the latter of which later became known as Orai, named after the keeper of heaven’s gate in Greek mythology [11,12] (Box 1).

Box 1 **Store-Operated Ca^2+^ Entry (SOCE).**

- SOCE consists of ER STIM1 and plasma membrane Orai1 [11,12]
- STIM1 consists of an EF hand which senses ER store depletion [12]
- Orai1 represents the pore-forming subunit of SOCE [13]
- ER store depletion triggers STIM1 oligomerization
- STIM1 oligomers activate Ca^2+^ entry through Orai1 channels
- SOCE channels consist of either Orai1 homotetramers or Orai1-Orai3 heterotetramers

The transient receptor potential (TRP) channels, named after the Drosophila mutants with a defective visual transduction system (transient light-induced photoreceptor depolarisation response), represent a large and diverse family of Ca^2+^-permeable, non-selective ion channels [14]. TRP channels are subdivided into TRPC (TRPC1–C7), TRPV (TRPV1–6), TRPM (TRPM1–8) and the more obscure families of muculipins (TRPML), polycystins (TRPP1–2), ANKTM, and the list continues to grow [14]. TRP channels function primarily as cellular sensors of the environment. This includes temperature (TRPV1–4, TRPM8), TRPV1 senses heat and capsaicin [15] and TRPM8 senses cold and menthol); osmolality/pain (TRPV4); pheromone sensing in rodents (mTRPC2 [16]); redox/metabolism (TRPM2 [17]); and mechanical forces, cell volume, membrane stretch, and ATP depletion (TRPM7).

Ca^2+^ clearance pathways primarily include the ATP-driven Ca^2+^ pumps found on the plasma membrane (plasma membrane Ca^2+^ ATPase, PMCA) [18], sarco/endoplasmic reticulum (sarco/endoplasmic reticulum Ca^2+^ ATPase, SERCA) and golgi apparatus or secretory pathway (secretory pathway Ca^2+^ ATPase, SPCA) [19]. Additional Ca^2+^ clearance pathways include the Na^+^/Ca^2+^ exchanger (NCX) [20], which is driven by the inward electrochemical Na^+^ gradient/membrane potential, and the mitochondrial Ca^2+^ uniporter (MCU) responsible for Ca^2+^ uptake into the mitochondria [21].

## 4. Ca^2+^ Signalling in Key Cancer Processes

### 4.1. Cell Proliferation 

**Figure 2:**
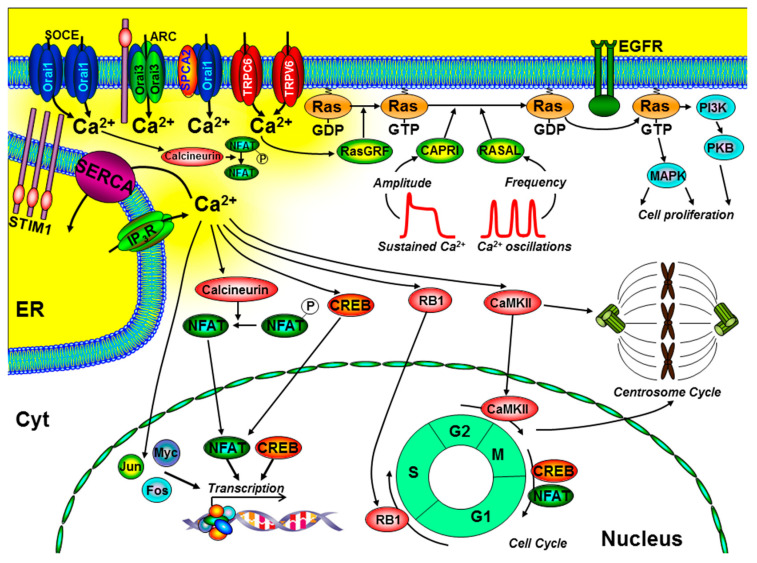
The role of key components of the Ca^2+^ signalling machinery in the control of cell proliferation and the cell cycle. Ca^2+^ entry through store-operated Ca^2+^ channels (SOCE; consisting of endoplasmic reticulum (ER) stromal interacting molecule (STIM)1 and Orai1), arachidonate-regulated Ca^2+^ channels (ARC; consisting of plasma membrane STIM1 and Orai1/Orai3 heteropentamers), transient receptor potential channel (TRPC)6, TRPV6, and the non-SOCE (plasma membrane secretory pathway ATPase (SPCA)-regulated Orai1) have all been implicated to regulate cell proliferation. Specific spatiotemporal patterns of Ca^2+^ signals can differentially regulate gene transcription (via calcineurin/nuclear factor of activated T cells (NFAT), CAMKII/cAMP response element-binding protein (CREB), immediate early genes (Jun, Myc, and Fos)), and the Ras/ERK signalling pathway. Localised Ca^2+^ entry can specifically activate the Ras guanine exchange factor (Ras-GEF), Ras-GRF, which converts the inactive Ras-guanosine diphoshpate (Ras-GDP) to the activate Ras-guanosine triphoshpate (Ras-GTP). Moreover, the amplitude of Ca^2+^ signals can specifically activate CAPRI and the frequency of Ca^2+^ oscillations can specifically activate RASAL, both of which are Ras guanine activating proteins (Ras-GAPs) that inactivate Ras-GTP. Ca^2+^ also has an important role in the control of the cell cycle and centrosome cycle. Specifically, Ca^2+^-dependent activation of retinoblastoma-1 (RB1) regulates G1/S phase transition, CaMKII regulates G2/M phase transition, and CREB and NFAT can regulate M/G1 transition. Additional abbreviations include EGFR, epidermal growth factor receptor; PI3K, phosphinositide-3 kinase; PKB, protein kinase-B; MAPK, mitogen-activated kinase; cell cycle phases: G1 and G2, gap; S, synthesis; M, mitosis.

Ca^2+^ signalling can activate several key signalling pathways and transcription factors that control cell proliferation and the cell cycle (Figure 2). Most notably, these include the transcription factors, nuclear factor of activated T cells (NFAT) [22], Oct/OAP, NFκB [5,6], cAMP response element-binding protein (CREB) [23], immediate early genes (FOS, JUN, and MYC) [24,25], as well as activation of the Ras-extracellular-signal-regulated kinase (Ras-ERK) pathway [26]. Importantly, many of these Ca^2+^-dependent effectors can be differentially regulated by specific spatiotemporal Ca^2+^ signals (see Figure 2). Localised Ca^2+^ entry can specifically and efficiently activate Ras-ERK pathways via Ca^2+^/calmodulin (CaM) and guanine nucleotide exchange factors (GEFs) [27,28]. In addition, the frequency of Ca^2+^ oscillations can differentially regulate different transcription factors [5,6] and Ras/ERK pathways, via the Ras-guanine activating proteins (GAPs), CAPRI, and RASAL [29,30]. These are all important steps in the control of cell proliferation (Figure 2).

Ca^2+^ also has an important role at various stages of the cell cycle, especially early G1, G1/S transition, and progression through mitosis [31]. Ca^2+^-dependent phosphorylation of retinoblastoma-1 (RB1) is one of the key regulatory steps at the G1/S boundary and CaM kinase (CaMK) regulates progression through G1 and mitosis [24,25]. Moreover, the Ca^2+^-dependent phosphatase, calcineurin, is important during G1 and S phases, owing to the activation of the Ca^2+^-dependent transcription factors, CREB [23] and NFAT [22], which regulate the expression of key cyclin family of proteins (cyclin A, E, and D1) and cyclin-dependent kinases (CDK2, CDK4) [31]. Incidentally, specific Ca^2+^ entry through Orai1 [32], TRPC6 [33], and TRPV6 [34] has been shown to specifically and efficiently regulate NFAT-mediated gene expression. Furthermore, Ca^2+^ and CaMKII have been reported to directly regulate centrosome duplication and separation, which, when defective, can lead to aberrant mitotic spindles, aneuploidy, and genetic instability, all hallmarks of cancer [31] (Figure 2).

Another important feature of cancer cells is their limitless replicative capacity [1]. Most normal cells stop dividing after about 50 cell divisions, owing to a shortening of their telomeres during successive cycles of replication. This phenomenon is known as cellular or replicative senescence and can trigger apoptosis if the DNA damage cannot be repaired. One way in which cancer cells can avoid senescence and acquire resistance to apoptosis is to up-regulate telomerase expression to maintain their telomeres. The EF-hand containing Ca^2+^-binding protein, S100A8, inhibits telomerase activity [35], thereby accelerating cellular senescence. This suggests that remodelling of Ca^2+^ signalling, and in particular attenuated Ca^2+^ signaling, likely contributes to cancer cell immortality.

### 4.2. Cell Death

**Figure 3:**
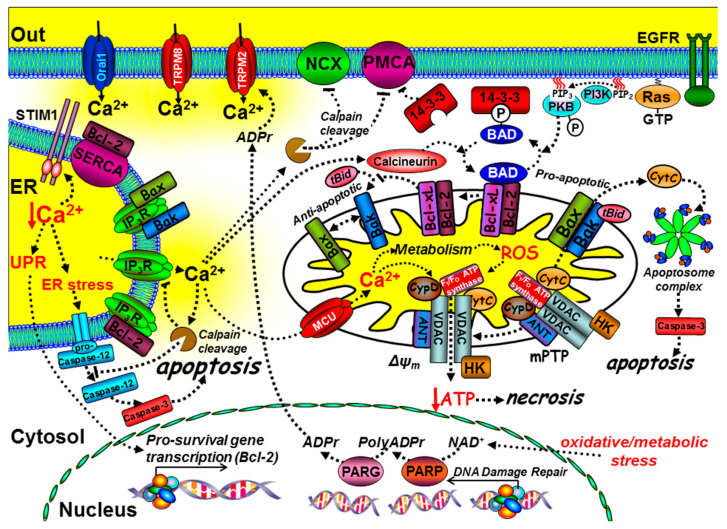
The role of key components of the Ca^2+^ signalling machinery in cell death and immortality. Ca^2+^ mediates intrinsic cell death at both the mitochondria and ER. At the mitochondria, tBid binds to and promotes Bax and Bak oligomerisation into pores that release cytochrome C, which binds to the apoptosome complex and activates the executioner caspases, such as caspase-3, leading to the “point-of-no-return” apoptotic cascade. The anti-apoptotic proteins, Bcl-2 and Bcl-xL, can prevent the t-Bid/Bax/Bak interaction, thereby preventing apoptosis. The pro-apoptotic protein Bad binds to Bcl-2/Bcl-xL, thereby preventing their interaction with the t-Bid/Bax/Bak complex, thus promoting apoptosis. Phosphorylation of Bad, via growth factor receptor-mediated activation of protein kinase-B (PKB), causes Bad to dissociate from the mitochondria and bind to 14-3-3 protein. Sustained Ca^2+^ overload can activate calcineurin, which dephosphorylates Bad, allowing it to sequester the anti-apoptotic proteins, Bcl-2/Bcl-xL. Ca^2+^ uptake into the mitochondria, via the mitochondrial Ca^2+^ uniporter (MCU), can lead to the production of reactive oxygen species (ROS). Ca^2+^ and ROS can activate the permeability transition pore (mPTP), loss of the mitochondrial membrane potential (ΔΨm), ATP depletion and necrosis. Ca^2+^ overload can also activate calpain and cleavage of the PMCA, Na^+^/Ca^2+^-exchange (NCX), and inositol 1,4,5-trisphosphate receptors (IP3Rs). Bax, Bak, and Bcl-xL can also bind to and inhibit IP_3_Rs. Bcl-2 can bind to and inhibit SERCA, reducing ER Ca^2+^.

Ca^2+^ has a critical, yet paradoxical role in regulating cell death [36] and, in particular, both ER Ca^2+^ and mitochondrial Ca^2+^ are central to this (Figure 3). Cancer cells adopt strategies to avoid cell death by activating pro-survival pathways and suppressing cell death machinery. The main regulators of cell death include the pro- and anti-apoptotic B-cell lymphoma 2 (Bcl-2) family of proteins; there are currently around 20 different members of this family, though the list continues to grow [37]. The balance between pro-apoptotic (Bax, Bak, and Bad) and anti-apoptotic proteins (Bcl-2/Bcl-xL) determines whether a cell is sensitive or resistant to apoptosis [38] (Figure 3 and Box 2). The phosphorylation status of Bad is a critical checkpoint for apoptosis (Figure 3 and Box 2).

Box 2 **Cell Death.**

***Pro-apoptosis***

- Bax-Bak oligomerisation induces outer mitochondrial membrane permeabilization (OMM) [39]
- OMM permeabilisation causes cytochrome C release
- Cytochrome C associates with apoptosome complex and activates executioner caspases [36,40]
- t-Bid promotes Bax and Bak oligomerisation and cytochrome C release [39]
- Bad binds to and inhibits the anti-apoptotic proteins Bcl-2 and Bcl-xL [41,42]
- Calcineurin dephosphorylates Bad allowing it to bind to Bcl-2 and Bcl-xL [43]

***Anti-apoptosis***

- Bcl-2 and Bcl-xL inhibits Bax and Bak oligomerisation and cytochrome C release [44]
- PKA [45], MAPK [46] and PKB [47] phosphorylate Bad
- Phosphorylated Bad dissociates from the mitochondria and binds to 14-3-3 protein
- Growth factor signalling leads to phosphorylation of Bad

When phosphorylated by protein kinase-A (PKA), protein kinase-B (PKB), or Ras-mitogen activated kinase (MAPK), Bad dissociates from the mitochondria to the cytosol, where it binds to 14-3-3 protein, which prevents it from engaging and inhibiting the anti-apoptotic Bcl-2/Bcl-xL; this ultimately inhibits apoptosis [42] (Figure 3 and Box 2). On the other hand, the Ca^2+^-dependent activation of calcineurin leads to the dephosphorylation of Bad, allowing it to bind to and inhibit Bcl-2/Bcl-xL, thereby promoting apoptosis [43]. It is also interesting to note that 14-3-3 proteins have been reported to inhibit the PMCA [48], thereby accentuating the Ca^2+^/calcineurin-mediated dephosphorylation of Bad and further potentiating this Ca^2+^-mediated apoptosis. Therefore, a remodelling of the Ca^2+^ signalling machinery that leads to “dampening” of Ca^2+^ signals can result in apoptosis resistance.

In addition, Ca^2+^ has a more prominent role during cell stress-induced cell death [36] (Figure 3). Mitochondria take up Ca^2+^ via a low affinity mitochondrial Ca^2+^ uniporter (MCU) [49,50]. MCU is important for the activation of a number of key metabolic enzymes [51], a process referred to as stimulus-metabolism coupling. Owing to the low affinity of the MCU, many mitochondria are strategically positioned in close proximity to either Ca^2+^ entry channels or Ca^2+^ release channels in order to maximise mitochondrial Ca^2+^ uptake [52]. Some studies have shown that this is a dynamic process to meet the energy demands of the cell [53,54]. However, sustained and excessive cytosolic Ca^2+^ overload can lead to excessive mitochondrial Ca^2+^ uptake and an elevated mitochondrial matrix Ca^2+^ concentration, which in turn can lead to the production of reactive oxygen species (ROS) [55]. This is a common phenomenon in many diseases in which there is Ca^2+^-mediated and/or oxidative stress-dependent cell death or cellular injury. However, down-regulation of MCU expression, mediated by the over-expression of the cancer-related MCU-targeted microRNA, miR-25, has been reported to contribute to apoptosis resistance in colon cancer [56]. In addition, ROS have multiple and complex effects on both pro-survival pathways and the Ca^2+^ signalling machinery. For example, ROS can induce the cleavage and thus inactivation of PKB [57], yet it can also oxidise and inactivate phosphatidylinositol-3,4,5-trisphosphate (PIP_3_) 3-phosphatase (PTEN), which paradoxically increases PKB activity [58]. ROS can amplify receptor tyrosine kinase (RTK) signalling, via activation of PLCγ and IP_3_ production [59]. Furthermore, ROS can directly sensitize Ca^2+^ release channels [60] and activate TRPM2 channels [61]. Both ROS and Ca^2+^ can activate the mitochondrial permeability transition pore (mPTP) [55], which consists of the voltage-dependent anion channel (VDAC), adenine nucleotide translocase (ANT), and cyclophilin-D [62] (Figure 3). This molecular machine is responsible for coupling mitochondrial volume and ion homeostasis with metabolism and cellular stress (see Box 3). The mPTP sits at the inner mitochondrial membrane (IMM)–outer mitochondrial membrane (OMM) interface; excessive activation thus leads to mitochondrial swelling owing to impaired mitochondrial ion homeostasis (Box 3) and OMM rupture, which can also cause the release of cytochrome C (Figure 3) [36].

Box 3 **Ca^2+^ and Ion Homeostasis.**

Ca^2+^ is inextricably linked to cytosolic ion homeostasis, cell volume regulation and intracellular pH regulation as well as mitochondrial ion homeostasis and volume regulation. These have all been separately implicated in the regulation of numerous cellular functions including the key cancer phenotypes of cell migration, invasion, cell proliferation and cell death. There are numerous Ca^2+^-dependent ion channels, such as Ca^2+^-dependent K+ channels (e.g., BKCa, IKCa, SKCa), Ca^2+^-dependent Cl- channels (e.g., TMEM16A) that are expressed on the plasma membrane and also the mitochondrial membrane. Ca^2+^ flux through Ca^2+^ channels and the activation Ca^2+^-dependent ion channels on either the plasma membrane or mitochondrial membrane can affect the membrane potential and thus the driving force for most ion channels and transporters in that membrane.

**Figure 4:**
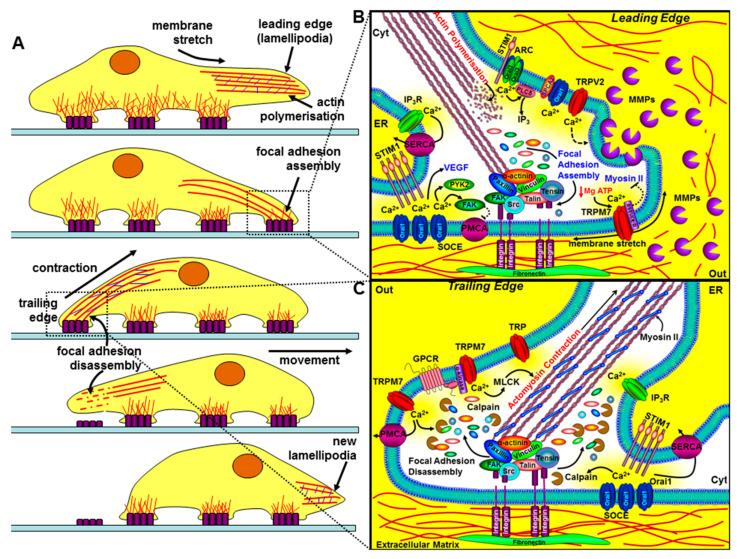
The role of key components of the Ca^2+^ signalling machinery in cell migration and invasion. The left panel (**A**) depicts the sequential steps of a migrating cell. The bottom part of the cell makes contact with the extracellular matrix (ECM) via integrins (purple rectangles), which couple the ECM to the actin cytoskeleton. The leading edge involves specialised membrane protrusions (lamellipodia/invadapodia) containing matrix metalloproteinases (MMPs), actin polymerisation, the assembly of focal adhesions, and the formation of new integrin-mediated contact sites. The trailing edge requires focal adhesion disassembly, actomyosin contraction, and the loss of rear-end contact sites. The right panels (**B** and **C**) show a magnified view of how key components of the Ca^2+^ signalling machinery regulate the leading edge (**B**) and the trailing edge of a migrating cell (**C**). At the leading edge, specific localised Ca^2+^ entry, amplified by Ca^2+^ release through IP_3_Rs, can lead to focal adhesion assembly via the Ca^2+^-dependent activation of calmodulin-dependent kinase (CaMKII), proline-rich tyrosine kinase (PYK2), and focal adhesion kinase (FAK). Ca^2+^ entry through store-operated Ca^2+^ channels (SOCE; consisting of ER STIM1 and Orai1), TRPM7, TRPV2, arachidonate-regulated Ca^2+^ channels (ARC; consisting of plasma membrane STIM1 and Orai1/Orai3 heteropentamers), and the non-SOCE (plasma membrane SPCA-regulated Orai1) have all been implicated in the regulation of cell migration and invasion. Membrane stretch can activate Ca^2+^ entry through TRPM7 and the α-kinase domain of TRPM7 can also phosphorylate myosin-IIA heavy chain and inhibit actomyosin contraction and lead to cell spreading. Ca^2+^ entry through TRPV2 has also been shown to regulate the release of MMPs and the focal degradation of the ECM. At the trailing edge, Ca^2+^ entry through SOCE and TRPM7 channels leads to the coordinated Ca^2+^-dependent activation of m-calpain, leading to focal adhesion disassembly, and myosin light chain kinase activation, leading to actomyosin contraction.

For many years, the mPTP was thought to be important for Ca^2+^-mediated apoptosis [63,64]; however, knockout studies of VDAC [65] and cyclophilin-D [66,67] had no effect on apoptosis, but attenuated necrotic cell death. This suggests that the mPTP may be more important during necrotic cell death than for apoptosis. Nevertheless, it is clear that the mPTP can functionally interact with Bcl-2/Bcl-xL and Bax/Bak [63,64,68,69], suggesting that necrosis and apoptosis may share some of the same molecular machinery. The defining feature of necrosis is that this is usually accompanied by the collapse of the mitochondrial membrane potential, owing to excessive activation of the mPTP and the consequent inhibition of mitochondrial ATP synthesis. This can have a knock-on effect on Ca^2+^ homeostasis, specifically owing to the inhibition of the ATP-dependent Ca^2+^ pumps (PMCA and SERCA), but also inhibition of the Na^+^/K^+^-ATPase, which is important for maintaining the plasma membrane potential, and thus the driving force for Ca^2+^ entry (Box 3 and Figure 4). Clearly, enhanced Ca^2+^ signalling and Ca^2+^ overload promote Ca^2+^-mediated cell death (necrosis and apoptosis). However, there is extensive evidence that remodelling of the Ca^2+^ signalling machinery that leads to an overall “dampening” of Ca^2+^ signals can result in apoptosis resistance, an important hallmark of cancer (Figure 3).

In addition to mitochondrial Ca^2+^ content, cell death pathways are also influenced by ER Ca^2+^ content. ER Ca^2+^ concentration is critical for the correct synthesis, folding, and processing of newly synthesised proteins. Perturbation of ER Ca^2+^ can lead to either accumulated protein or incorrectly folded proteins, resulting in ER stress or the unfolded protein response (UPR) [70]. This leads to the activation of numerous ER chaperone proteins and transcriptional control of pro-survival genes (e.g., Bcl-2/Bcl-XL) and inhibition of the protein translation machinery, both of which are generally regarded as cytoprotective. However, prolonged and excessive ER stress or cytosolic Ca^2+^ overload can lead to the activation of m-calpain and the cleavage of pro-caspase-12, which normally resides on the ER membrane [71]. The cleavage product (caspase-12) can then activate downstream executioner caspases that engage a “point of no return” apoptosis cascade [71]. Ca^2+^ can also amplify cell death by activating cytosolic calpains [72], which can directly activate caspases [73] and inactivate the anti-apoptotic Bcl-2 [74], the Na^+^/Ca^2+^-exchange (NCX), and the PMCA [75,76,77,78,79] (Figure 3).

Paradoxically, some of the pro-apoptotic and anti-apoptotic proteins have been reported to directly regulate key Ca^2+^ transport proteins including IP_3_Rs [80,81], SERCA [82], PMCA [83], and MCU [84], resulting in a complex reciprocal regulation between Ca^2+^ signalling and cell death. Specifically, cytochrome C, once released from mitochondria, can bind to and potentiate Ca^2+^ release from IP_3_Rs [85], suggesting a feed-forward potentiation of apoptosis. Bax and Bak can regulate IP_3_Rs [86,87]. Bcl-2 is reported to reduce ER Ca^2+^ [80,81] and directly inhibit IP_3_Rs [88] and SERCA [82], which leads to reduced Ca^2+^ release and mitochondrial Ca^2+^ uptake. Although such remodelling of the Ca^2+^ signalling machinery may have evolved to attenuate Ca^2+^-mediated cell death, paradoxically, this may also result in apoptosis resistance (Figure 3).

Under metabolic or oxidative stress, free ADP-ribose is generated by the DNA repair machinery (poly (ADP-ribose polymerase (PARP1)/poly(ADP)-ribose glycohydrolase (PARG)) [89], resulting in the activation of Ca^2+^ entry through TRPM2, which likely contributes to the further activation of Ca^2+^-mediated cell death pathways [17] (Figure 3).

### 4.3. Migration and Invasion

Chemo-attractant-induced cellular migration involves the coordinated and dynamic regulation of structural and signalling linkages between the extracellular matrix and cytoskeleton, involving integrins, focal adhesion complexes [90], and the contractile machinery [91]. Accumulating evidence points to an increasingly important role of spatiotemporally distinct Ca^2+^ signals in directional sensing, redistribution of the actin cytoskeleton, traction force generation, and focal adhesion turnover [92]. Migrating cells exhibit a rear-to-front Ca^2+^ gradient responsible for the rear end retraction [93]. This is owing to the Ca^2+^-dependent activation of myosin light chain kinase (MLCK) and subsequent actomyosin contraction [94], and focal adhesion disassembly by Ca^2+^-dependent calpain cleavage of focal adhesion kinase (FAK), integrins, talin, and vinculin [95] (Figure 4). However, the leading edge of the migrating cells exhibit spatially confined Ca^2+^ oscillations, or “Ca^2+^ flickers”, that specifically activate focal adhesion assembly, and thus the growth and steering of the migrating cell [96,97]. This is owing to the specific Ca^2+^-dependent activation of CaMKII [98] and proline-rich tyrosine kinase-2 (PYK-2) [99], which leads to the tyrosine and serine phosphorylation of focal adhesion kinase (FAK), respectively. It is also important to note that activation of FAK has been reported to inhibit PMCA4b in platelets [100], which, if extrapolated, might accentuate localised Ca^2+^ signals at the leading edge of the migrating cell (Figure 4).

In the case of cancer cells, the leading edge is often referred to as the “invadopodia”—membrane protrusions that contain proteolytically active matrix metalloproteinases (MMPs) to focally degrade the extracellular matrix (ECM) [101] (Figure 4). Furthermore, the EF hand Ca^2+^-binding protein, S100A4, has been shown to translocate from the nucleus to the plasma membrane in response to an increase in cytosolic Ca^2+^ in migrating cancer cells [102]. S100A4 has been implicated in metastasis of cancer cells by interacting with non-muscle myosin IIA, IIB, and tropomyosin, thereby indirectly regulating migration [103]. Moreover, S100A4 has been shown to increase the expression of MMP9, leading to the proteolytic cleavage of the ECM and an increased invasive phenotype [104].

There are numerous mechano-sensitive Ca^2+^-permeable channels that sense membrane stretch at the leading edge of migrating cancer cells (Box 4).

Box 4 **Mechanosensitive Ca^2+^ Channels and Cancer.**

- Piezo1 and 2 are bona fide mechanosensitive non-selective Ca^2+^ permeable cation channels stimulated by all types of mechanical stimuli to the plasma membrane [105,106,107,108]
- Piezo channels are overexpressed in gastric, breast, prostate and glioma where they regulate cell proliferation, migration/invasion and angiogenesis but are oncosuppressors in lung and osteosarcoma [106]
- TRPM7 is a bi-functional stretch-activated Ca^2+^ and Mg^2+^ permeable channel [109,110]
- TRPV2 is a heat/mechano-sensitive Ca^2+^/Mg^2+^ permeable channel expressed in human leukemia cells [111]
- Panexins (Panx1) are mechanosensitive channels permeable to ATP which activate P2X7 purinergic ATP-gated Ca^2+^ permeable channels both of which are important in cancer [112,113]
- Polycyctins (TRPP1, TRPP2) are multi-functional mechanosensitive Ca^2+^ permeable channels that are over-expressed and facilitate an aggressive phenotype in colorectal cancer [114]
- TRPC1, TRPC2, TRPV1, TRPM3, TRPM4, TRPA1 also exhibit mechanosensitivity [113,115]

Arguably one of the most studied and potentially important of these stretch-activated Ca^2+^ channels involved in migration in non-cancer cells is TRPM7. This bi-functional Ca^2+^ and Mg^2+^ permeable channel, with a functionally separate regulatory α-kinase domain, can be activated by mechanical force, phospholipase-C (PLC)-coupled agonists, and Mg-ATP depletion [110] (Figure 4 and Box 5).

Box 5 **TRPM7 and Cellular Migration.**

- TRPM7 is a bi-functional stretch-activated Ca^2+^ and Mg^2+^ permeable channel [109,110]
- TRPM7 contains a functionally separate α-kinase domain [109,110]
- TRPM7 is activated by mechanical force, PLC-coupled agonists and Mg-ATP depletion [109,110]
- TRPM7 facilitates spatially confined Ca^2+^ signals at the leading edge of migrating cells [96,116]
- Ca^2+^ entry through TRPM7 activates calpain cleavage of focal adhesions [117]
- The TRPM7 α-kinase domain inhibits actomyosin contraction and facilitates cell spreading [118]

### 4.4. Angiogenesis

Cancer cells secrete a variety of growth factors, most notably vascular endothelial growth factor (VEGF), which have potent mitogenic and angiogenic effects on adjacent endothelial cells, causing cell proliferation, tube formation, and thus growth of new blood vessels [119]. Ironically, the mitogenic and angiogenic effects of VEGF in endothelial cells are mediated by Ca^2+^ signalling [120]. Most notably, Orai1 and STIM1 have been shown to mediate VEGF-induced endothelial cell migration, tube formation, and angiogenesis [120]. In addition, various TRP channels have been implicated in agonist and stretch-induced Ca^2+^ entry-mediated angiogenesis and microvascular permeability [119].

## 5. Opportunities for Targeting the Ca^2+^ Signalling Machinery in Cancer

There is extensive evidence that the expression of numerous Ca^2+^ channels and transporters is altered in many different types of cancer; in most cases, this is an over-expression, but in some cases, down-regulation, with both potentially leading to tumorigenesis and cancer progression (Table 1).

However, despite the extensive remodelling of the Ca^2+^ signalling machinery that occurs during cancer, it is very difficult to determine whether a change in expression is the cause or consequence of cancer progression. In addition, up-regulation of Ca^2+^ channels and transporters does not guarantee that these would necessarily represent ideal therapeutic targets, especially when one considers the ubiquitous nature of Ca^2+^ signalling. Therefore, any drug designed to target such a Ca^2+^ channel/transporter would be expected to have adverse effects, regardless of the specificity of such a drug. Therefore, an ideal therapeutic target would be one that is either uniquely expressed in cancer or whose expression leads to a totally novel function in cancer cells.

### 5.1. TRP Channels

Several TRP channels are reported to be overexpressed in cancer (Table 1). To date, the most well studied TRP channel in this context is TRPM7, a Ca^2+^-permeable nonselective cation channel with a kinase domain. TRPM7 has a role in mechanosensory signalling and the regulation of cellular contractility, adhesion, and migration. TRPM7 expression is elevated in ovarian [165], pancreatic [166,167], bladder [168], and breast [169] cancers, with high expression correlated with tumour growth, metastasis, and poor prognosis (see Box 5).

Moreover, TRPM7 has been linked to epithelial–mesenchymal transition (EMT, a key process underlying cancer cell motility and metastasis) in numerous cancers. In breast cancer cells, TRPM7 influences EMT by regulating the transcription factor SOX4 via changes in cell tension [170]. TRPM7 has been linked with EMT in ovarian [171] and bladder [172] cancers, the former by calcium-dependent regulation of the PI3K/AKT pathway, and appears to regulate transforming growth factor-β (TGFβ)-induced cell migration and EMT in prostate cancer cells [173]. In nasopharyngeal carcinoma cells, bradykinin-induced stimulation and over-expression of TRPM7 increased migration, whereas inhibition (with La^3+^ or 2-APB) and RNAi silencing of TRPM7 inhibit migration [143]. In human lung adenocarcinoma A549 cells, enhanced surface expression of TRPM7 contributes to both basal and EGF-stimulated migration [174]. Nuclear accumulation of myocardin related transcription factor-A in hepatocellular carcinoma cells (a known regulator of oncogenic transformation) was inhibited by TRPM7 channel blockade with NS8593 or knockdown by siRNA [175]. Interestingly, blockade of the TRPM7 kinase domain inhibited its regulation of focal adhesions and the cytoskeleton and resulted in inhibited cell migration in MDA-MB-231 cells [176], indicating a role for the TRPM7 kinase domain in metastasis. Moreover, hypoxia-induced ATP depletion stimulates TRPM7 activity, which facilitates cancer cell migration via activation of m-calpain and focal adhesion turnover [177].

In addition to being an attractive therapeutic target, TRPM7 is also a good prognostic marker for breast cancer. Tumour TRPM7 mRNA expression correlates with the incidence of reoccurrence and metastasis in breast cancer patients [178]. Xenograft models of human breast cancer revealed that increased TRPM7 expression led to increased invasiveness and metastasis [178]. Furthermore, TRPM7 has a 13-fold higher expression in pancreatic ductal adenocarcinoma (PDAC) compared with non-cancer tissue, with TRPM7 expression correlated with tumour progression and a poor survival rate [142]. Interestingly, the observation in this study that siRNA silencing of TRPM7 inhibits BxPC-3 cell migration without affecting proliferation suggests that TRPM7 specifically regulates the invasive phenotype of PDAC [142]. Furthermore, TRPM7 has recently been identified as a regulator of SOCE, despite not being a store-operated channel itself [179], with implications for crosstalk between TRPM7 channels and SOCE in cancer.

Collectively, these studies suggest that TRPM7 may represent a good therapeutic target to prevent a metastatic cancer cell phenotype. However, while the TRPM7 activator naltriben appears to enhance the invasive behaviour of glioblastoma multiforme cell line U87 [180] and TRPM7-selective inhibitors (Waixenicin A) that have been described [181,182], it remains to be determined whether pharmacological blockade of TRPM7 has any cancer-specific therapeutic potential in the clinic.

Beyond TRPM7, other melastatin-related TRP family receptors with putative roles in cancer progression are TRPM1 and TRPM8. TRPM1 expression appears to be inversely correlated to an aggressive metastatic melanoma phenotype [183] and has been described as a tumour suppressor. Over-expression of TRPM8 in PC-3 prostate cancer cells induced cell cycle arrest, facilitated starvation-induced apoptosis, and reduced migration, owing to the inactivation of focal adhesion kinase (FAK) [184]. More recent studies have shown that androgen receptors regulate prostate cancer cell migration via inhibition of TRPM8 and lowering of [Ca^2+^]_i_ [185]. This raises the possibility of using a TRPM8 activator to inhibit metastatic cells. Indeed, the classical TRPM8 agonist menthol has been shown to inhibit cell proliferation, induce cell cycle arrest, and inhibit migration of the androgen-independent DU145 prostate cancer cell line [186].

Transient receptor potential vanilloid 6 (TRPV6) is another TRP channel that has been suggested as a target for cancer [187]. TRPV6 is overexpressed in numerous solid tumours, including breast, colon, ovarian, prostate, and thyroid carcinomas [156], and is associated with poor prognosis [188]. The aberrant cell surface expression of TRPV6 in prostate cancer is increased in prostate cancer cells via SOCE-dependent activation of the Annexin I/S100A11 complex [189]. Moreover, this TRPV6 overexpression confers apoptosis resistance and aggressive behaviour in vivo [189]. Similarly, TRPV6 silencing in breast cancer cells with increased endogenous TRPV6 led to lower basal calcium influx and reduced proliferation [190]. Elevated TRPV6 is associated with poor prognosis in pancreatic cancer; moreover, knockdown of TRPV6 decreased Bcl-2 and increased BAX in Capan-2 cells, inhibiting proliferation and migration, while promoting apoptosis [191]. Interestingly, in human small cell lung carcinoma cells, the classical TRPV1 agonist capsaicin induces apoptosis via TRPV6, independent of TRPV1, in a Ca^2+^- and calpain-dependent manner [192]. These advances have led to preclinical and clinical studies assessing TRPV6 as a therapeutic target; the selective TRPV6 inhibitor SOR-C13 has shown promise in both in vivo models of cancer [193] and early clinical trials in patients with advanced tumours of epithelial origin [194].

Another TRPV channel involved in regulation of breast cancer cell angiogenesis and migration is TRPV4. Evidence from endothelial cells (ECs) derived from human breast carcinomas suggests that arachidonate-induced intracellular Ca^2+^ signals mediated by TRPV4 contribute to tumour angiogenesis [195]. Knockdown of TRPV4 decreases breast cancer cell invasiveness in vitro and lung metastasis in vivo [196], and further investigation revealed that TRPV4 promotes breast cancer metastasis via Ca^2+^-dependent activation of AKT and downregulation of E-cadherin expression [197]. Interestingly, a recent clinical study in patients with stable heart failure found that the novel, first-in-class TRPV4 inhibitor GSK2798745 was well tolerated, suggesting that TRPV4 blockade might be a safe target for metastatic breast cancer [198]. However, conflicting evidence suggests that downregulation of TRPV4 in tumour endothelial cells leads to increased tumour angiogenesis and enhanced tumour growth in vivo [199], and that activation (rather than inhibition) of TRPV4 promotes cell death and inhibits in vivo tumour formation in certain breast cancer cell lines [188]. These contrasting results highlight the challenge with targeting these channels in a heterogeneous tumour, and suggest that TRPV4 can have both pro- and antitumorigenic properties.

Alongside TRPV6 and TRPV4, TRPV2 is a further vanilloid family receptor that has been implicated in cancer progression. Over-expression of TRPV2 in androgen-dependent LNCaP prostate cancer cells led to the induction of invasive markers, matrix metalloproteinases (MMP9) and cathepsin-B [153]. In addition, siRNA silencing of TRPV2 reduced the growth and invasion of PC-3 cells in xenograft models of prostate cancer and reduced the expression of invasive markers, MMP2, MMP9, and cathepsin-B [153], suggesting that inhibition of TRPV2 may reduce the aggressive phenotype of prostate cancer.

Our understanding of how other TRP channels might contribute to cancer progression is more limited. TRPM2 has been implicated in facilitating AKT-dependent migration and tumour formation in gastric cancer cells [200]. Elevated TRPC5 expression has been identified in colon tumours and metastases, where it is correlated with poor prognosis [201]; TRPC5 overexpression in colon cancer cells led to altered intracellular Ca^2+^ levels, decreased E-cadherin, and promotion of EMT, resulting in increased proliferation and invasion [202]. TRPC6-mediated elevations in [Ca^2+^]_i_ have been implicated in the development of multi-drug resistance in hepatocellular carcinoma cells [203]. More recent studies have described novel selective TRPC6 inhibitors that demonstrated efficacy in xenograft models of gastric cancer [204]. Furthermore, other TRP channels not yet directly correlated with cancer are known to be involved in mechanotransduction during cell migration, and thus could be involved in metastasis ([205] and Box 4). For example, TRPC1, TRPC2, TRPV1, TRPM3, TRPM4, and TRPA1 are all reported to exhibit mechanosensitivity [47,115]. Furthermore, the heat sensitive TRPV2, expressed in human leukemia cells, is also reported to be mechano-sensitive [111], and TRPP1/TRPP2 are over-expressed in colorectal cancer and facilitate an aggressive phenotype [46]. Therefore, more TRP channels may emerge as attractive therapeutic targets in the future as their role in cancer progression is further delineated.

### 5.2. SOCE

SOCE was first linked to tumour cell migration and metastasis in MDA-MB-231 breast cancer cells, where [135] siRNA-induced silencing of Orai1 or STIM1, or treatment with the SOCE blocker SKF96365, reduced in vitro migration and invasion [135]. Furthermore, Orai1 or STIM1 siRNA and SKF96365 inhibited lung metastasis following tail vein injection of MDA-MB-231 cells into severe combined immunodeficient (SCID) mice [135]. The mechanism for the Orai1/STIM1-induced migration and invasive properties was shown to be the result of focal adhesion turnover that was dependent on H-Ras and Rac1 [135]. Since this study, numerous studies have implicated SOCE in migration, EMT, and invasion in a diverse range of malignancies, including gastric cancer [206], colorectal cancer [207,208], melanoma [209], clear cell renal cell carcinoma [124], prostate [210], and hepatocellular carcinoma [211]. Intriguingly, numerous cancer-related Orai mutants have been identified from the cBioPortal tumour database, which result in constitutive Ca^2+^ entry and NFAT activation [212]. Furthermore, studies suggest that Orai1 can also functionally couple with other channels when promoting migration, with Orai1 in colon cancer cells forming part of a functional channel complex with small conductance Ca^2+^-activated potassium channel 3 (SK3) and TRPC1 channels to amplify SOCE, AKT activation, and migratory behaviour [213]. A similar association between SK3 and Orai1 has been observed in breast cancer cells, where it is mediated by SigmaR1, a stress-activated chaperone [207]. Interestingly, collagen I (a factor found in the tumour microenvironment) regulates colocalization of Orai1 and the Kv10.1 channel in breast cancer cells to enhance Ca^2+^ entry and cell survival [214,215], indicating that extracellular matrix factors within the tumour microenvironment can influence ion channel functional coupling to remodel SOCE in cancer cells.

Changes in STIM expression and function have also been implicated in cancer. Gene chip microarray of 295 breast cancer patients revealed a high STIM1 and low STIM2 expression in basal-like tumours that have a particularly poor prognosis and few treatment options. This suggests that the STIM1/STIM2 expression ratio may represent a good prognostic marker for breast cancer [122]. In addition, STIM1 has been shown to be important for cervical cancer cell proliferation, migration, and angiogenesis [134]. STIM1 expression was up-regulated in 71% of early stage cervical cancer and the level of expression in primary tumour tissue was linked to the occurrence of pelvic lymph node metastasis and poor patient survival [134]. STIM1 knockdown using shRNA and SOCE blockers (2-APB and SKF96365) reduced cervical cancer growth and the number of tumour blood vessels in cervical cancer xenograft mouse models [134]. This STIM1-induced migration was owing to Ca^2+^-dependent calpain and PYK2-mediated focal adhesion turnover, and the increase in angiogenesis was owing to STIM1-dependent secretion of VEGF from cervical cancer cells [134]. Conversely, in colorectal cancer cells, STIM1 regulates migration by stimulating the production of cyclooxygenase-2 and production of prostaglandin E2 [216].

Collectively, the above studies suggest that STIM1 and Orai1 represent good therapeutic targets and inhibitors of SOCE may reduce cancer cell proliferation, migration, invasion, and tumour angiogenesis. Indeed, novel SOCE blockers currently under development by CalciMedica and Rhizen Pharmaceuticals for other indications could be repurposed for cancer if successful. However, the ubiquitous nature of these proteins raises the possibility of adverse effects, regardless of the specificity of such drugs. Indeed, reduced Orai1 and STIM1 expression leads to apoptosis resistance in prostate cancer cells, presumably owing to reduced global Ca^2+^ overload [127], suggesting that prolonged pharmacological inhibition of SOCE would also likely lead to resistance to apoptosis. Thus, it remains to be determined whether any therapeutic benefit of blocking SOCE outweighs the detrimental effects of promoting apoptosis resistance.

### 5.3. Store-Independent Arachinodate-Regulated Ca^2+^ Channels (ARC)

In addition to the widely studied Orai1 and STIM1, there are additional isoforms including Orai2, Orai3, and Stim2 that contribute to SOCE [217,218]. Recent evidence shows that Orai3 is up-regulated in breast [123,129,130,131], lung [133], and prostate cancer [132], and contributes to tumorigenesis [129,130]. Furthermore, it has recently been discovered that the store-independent, arachidonate-regulated Ca^2+^ entry channel (ARC) [219] consists of heteropentameric subunits of Orai1 and Orai3 [220] (Box 6).

Box 6 **Arachidonate-Regulated Ca^2+^ Channels (ARC).**

- ARC is a store-independent, arachidonate-regulated Ca^2+^ entry channel (ARC) [219]
- ARC consists of heteropentameric subunits of Orai1 and Orai3 [220]
- ARC has similar molecular composition and biophysical characteristics to SOCE channels [219]
- Activation of ARC requires *plasma membrane* STIM1, rather than ER STIM1 [219]
- Ca^2+^ entry through ARC channels controls the frequency of Ca^2+^ oscillations
- Ca^2+^ entry through ARC channels activates PLC-δ and increases IP_3_ production [221]

**Figure 5:**
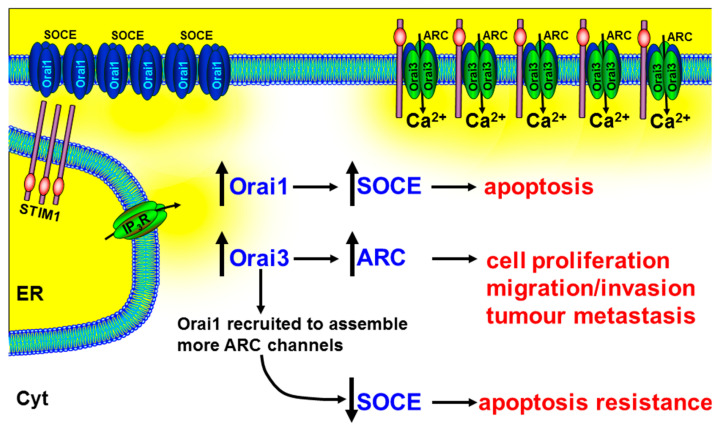
The role of Orai3 and ARC channels in numerous cancer hallmark responses. Arachidonate-regulated Ca^2+^ entry channels (ARC) consist of heteropentameric subunits of Orai1 and Orai3. The increase in Orai3 expression and/or change in the tumour microenvironment (arachidonic acid) lead to the recruitment of Orai1 subunits into the assembly of heteropentameric Orai1/Orai3 ARC channels and a corresponding decrease in available Orai1 subunits for the assembly of SOCE channels. The increase in the ARC channels leads to the NFAT-dependent cell proliferation, migration/invasion, and tumour metastasis, and the reduction in SOCE leads to apoptosis resistance. This suggests that drugs designed to specifically inhibit Orai3, or their assembly with Orai1, might inhibit tumour growth and metastasis, while simultaneously promoting apoptosis.

Therefore, the contribution of Orai3 in tumorigenesis could be owing to increased ARC channels. Indeed, it has been reported that the relative proportions of Orai proteins are altered in prostate cancer, compared with non-cancerous tissue, with a particularly up-regulated Orai3 expression [126]. Mimicking this up-regulated Orai3 expression in PC3 prostate cancer cell lines led to an increase in characteristic ARC-mediated, store-independent Ca^2+^ entry, and a consequent increase in NFAT-mediated cell proliferation [126]. A more recent study showed that arachidonic acid (AA) (or arachidonate)-regulated Ca^2+^-entry (ARC) induces migration in BON gastroenteropancreatic neuroendocrine tumour cells [222]. In addition, in xenograft models of prostate cancer, siRNA knockdown of Orai3 dramatically reduced tumour growth [126]. The authors speculated that the increase in Orai3 expression and/or change in the tumour microenvironment (arachidonic acid) led to the recruitment of Orai1 subunits into the assembly of heteropentameric Orai1/Orai3 ARC channels (Figure 5). In addition to increasing the ARC-mediated NFAT-dependent cell proliferation, this led to the reduction of Orai1 subunits for the assembly of homotetrameric Orai1-containing SOCE channels and the consequent apoptosis resistance [126] (Figure 5).

Another important feature of ARC channels is the regulation by plasma membrane STIM1 rather than ER STIM1 [219]. There is extensive evidence for the critical role of ER STIM1 in regulating SOCE. However, it is worth remembering that, prior to the discovery of the role of STIM1 in SOCE in 2005 [12,223], STIM1 was first identified in a screen for cell adhesion molecules [224], as its name suggests (stromal interacting molecule 1). Moreover, arachidonic acid is generated by activation of various growth factors [225] and has been implicated in the migration of breast cancer cells via FAK phosphorylation [226]. It is thus tempting to speculate that plasma membrane STIM1 and ARC channels may be important in cancer migration by sensing the tumour microenvironment. This could dramatically elevate the importance of ARC channels from relative obscurity to a critical role in cancer progression.

### 5.4. Secretory Pathway Ca^2+^-ATPase (SPCA)

Another way in which Ca^2+^ entry through Orai1 can be activated independent of STIM1 and store depletion is by the interaction of the secretory pathway ATPase-2 (SPCA2) with Orai1 in the plasma membrane, which was recently discovered to promote breast tumorigenesis [227]. The SPCA is expressed on the golgi and is important for the transport of Ca^2+^ and Mn^2+^ into the golgi lumen. These ions are important for the correct processing of newly synthesized proteins in the secretory pathway [228]. Evidence from breast cancer cell lines suggests that SPCA transporters might be the initiator of microcalcifications within breast tumours that correlate with tumour progression [229]. In basal-like breast cancer cells, SPCA1 is up-regulated and leads to an increase in the processing and trafficking of the insulin-like growth factor receptor (IGF-1R), which ultimately leads to an increased growth and proliferative phenotype [230]. This is perhaps not surprising when one considers that the normal function of SPCA is to regulate the synthesis and processing of proteins within the secretory pathway. However, SPCA2 is over-expressed in the plasma membrane of breast cancer cells, where it directly binds to and activates Orai1, independent of STIM1, and leads to enhanced Ca^2+^ entry and Ca^2+^-dependent proliferation [227]. Specifically, silencing SPCA2 in breast cancer cell lines reduced resting Ca^2+^, cell proliferation, anchorage-dependent growth, and breast tumour growth in mouse xenograft models, whereas over-expression of SPCA2 had opposite effects [227]. Interestingly, over-expression of a mutant SPCA2 without any ATPase activity also increased resting Ca^2+^ and anchorage-dependent growth, similar to the wild type SPCA2. This, therefore, suggests that the tumorigenic effects of SPCA2 were independent of its Ca^2+^-ATPase activity. Immunofluorescence and cell surface biotinylation assays revealed that SPCA2 partially localized to the plasma membrane and associated with the N-terminal domain of Orai1, where it directly regulated Ca^2+^ entry, led to the nuclear translocation of NFAT and increased cell proliferation. This mechanism is potentially an important drug target in malignant breast lesions, as SPCA is not normally expressed in the plasma membrane of normal cells. Therefore, targeting SPCA might be an effective therapeutic strategy for specifically treating breast cancer. Whether this SPCA2–Orai interaction and novel store-independent Ca^2+^ entry pathway are important in other types of cancer remains to be determined.

### 5.5. PMCA

PMCA has been shown to have an important role in cancer [157,158,231]. Overexpression of PMCA4 in PDAC correlates with poor prognosis, while siRNA knockdown of PMCA4 inhibited migration and apoptosis resistance in cultured PDAC cells [232]. In the breast, PMCA has an important role in the normal physiology and in tumorigenesis. PMCA2 is up-regulated during lactation and controls milk production in normal mammary epithelial cells, but is then down-regulated during weaning when the secretory cells die from apoptosis [233]. This loss of PMCA2 expression is thought to elevate resting cytosolic Ca^2+^, thereby increasing the sensitivity of Ca^2+^-dependent apoptosis [158] (Figure 3). In breast cancer, PMCA2 expression increases significantly; this eventually leads to resistance to apoptosis and is thought to contribute to tumorigenesis [158]. More recent studies have shown that, specifically, PMCA2 remodels [Ca^2+^]_i_ in breast cancer cells to facilitate continued HER2 biochemical signalling and PMCA2 knock out inhibits the formation of tumours in vivo [234]. However, direct inhibition of the PMCA is unlikely to be feasible as a therapeutic strategy owing to its ubiquitous expression. Targeting the pathophysiological coupling between calcium transporters and their downstream effectors might be a more subtle approach that would avoid toxicity.

## 6. Metabolic Regulation of the Ca^2+^ Transporters

Aberrant metabolism is a hallmark of cancer [235], with many transformed cells exhibiting a dramatic increase in glycolytic metabolism (the ‘Warburg effect’) that is key to maintaining a highly proliferative, pro-survival, and invasive cancer phenotype [236]. Remodelling of Ca^2+^ signalling is known to influence metabolic changes in cancer cells that in turn promote aggressive behaviour; for example, expression of MCU correlates with tumour size and metastasis in triple negative breast cancer, with evidence implicating mitochondrial Ca^2+^ uptake in promoting HIF-1α expression and invasiveness [237]. On the other hand, metabolism and the generation of ATP have a strong influence on Ca^2+^ signalling, and while cancer metabolism has been previously targeted for therapeutic intervention, as reviewed extensively elsewhere [238,239], the relationship between altered metabolism in cancer and its regulation of Ca^2+^ signalling remains relatively unexplored. Indeed, an alternative strategy to directly targeting the Ca^2+^ signalling machinery is to instead target those metabolic processes that regulate Ca^2+^ signalling, but are altered following metabolic transformation, thereby providing a means to selectively target malignant cells. Two of the main controllers of [Ca^2+^]_i_ homeostasis are the ATP-dependent Ca^2+^ pumps, PMCA and SERCA. These pumps are responsible for the maintenance of a low resting [Ca^2+^]_i_, and are inextricably linked to cell metabolism owing to their requirement for a robust supply of ATP [240]. Impairment of either PMCA or SERCA can lead to compromised [Ca^2+^]_i_ homeostasis with catastrophic consequences for any cell [241]. In many non-excitable cells, NCX is either absent or not functional; the PMCA is thus critical for Ca^2+^ efflux and its failure results in an inability to maintain a low resting [Ca^2+^]_i_, resulting in [Ca^2+^]_i_ overload and cell death [76,79,240]. Of relevance to cancer, the PMCA is critically reliant on glycolytically-derived ATP in pancreatic cancer cells exhibiting a highly glycolytic phenotype, with inhibition of glycolysis in these cells leading to ATP depletion, PMCA inhibition, Ca^2+^ overload, and ultimately necrotic cell death [242,243,244,245].

Evidence also suggests that the PMCA may be fuelled by a local, submembrane supply of glycolytically-derived ATP. Studies using inside-out smooth muscle plasma membrane vesicles established that glycolytic enzymes associate with the plasma membrane, thereby providing a submembrane pool of glycolytically-derived ATP to fuel the PMCA [246]. Extensions of this study indicate that PMCA preferentially uses ATP produced by these membrane-bound enzymes to fuel Ca^2+^ transport [247]. Silencing phosphofructokinase fructose bisphosphatase-3 (PFKFB3) at the membrane of endothelial cells prevents the generation of a sub-membrane glycolytic ATP pool and inhibits migration [248]. These findings highlight the broader potential importance of a sub-membrane glycolytic cascade in cell migration that could be extrapolated to cancer. More recent studies have determined that glycolytic enzymes, including PFKFB3 and the M2 isoform of pyruvate kinase (PKM2), associate with the plasma membrane in pancreatic cancer cell lines [244]. Moreover, specific pharmacological inhibition of either PKM2 [244] or PFKFB3 [245] inhibited PMCA activity, resulting in inhibition of migration and cytotoxic calcium overload. Given the aberrant changes in glycolytic enzyme expression prevalent in cancer cells, it is tempting to speculate that a privileged glycolytic ATP supply to Ca^2+^ pumps may be an untapped novel therapeutic locus. Owing to the aberrant expression of PFKFB3 and PKM2 in cancer cells compared with their corresponding “normal” cell counterparts, targeting these glycolytic enzymes might provide a means to target Ca^2+^ pumps specifically in cancer cells.

## 7. Calcium Signalling and the Tumour Immune Microenvironment

Beyond targeting Ca^2+^ signalling within cancer cells themselves, the tumour immune microenvironment offers an additional rich tapestry of potential targets for therapeutic intervention. Tumour-infiltrating immune cells exhibit their own repertoire of Ca^2+^ signalling machinery that has critical roles in their respective anti-tumour or pro-tumour functions. Moreover, the specific spatiotemporal pattern of Ca^2+^ signalling can differentially encode gene expression and cell fate in immune cells [5,6]. These immune cells include antigen-presenting dendritic cells (DCs), cytotoxic CD8+ T lymphocytes (CTLs), natural killer (NK) cells, type 1 and type 2 CD4+ T helper cells (Th1 and Th2), CD4+ regulatory T cells (Tregs), B lymphocytes, M1- and M2-like tumour-associated macrophages (TAMs), tumour-associated neutrophils (TANs), and myeloid-derived suppressor cells (MDSCs). Within any given tumour microenvironment, there is a balance between the “anti-tumour” activities of DCs, CTLs, NK cells, Th1 cells, and M1-TAMs, which cooperate to kill cancer cells, and the pro-tumour activities of M2-TAMs, Th2 cells, B cells, Tregs, and MDSCs, which simultaneously promote tumour progression and immune evasion [249,250,251]. This balance can vary dramatically depending on the tumour-type. It can also dynamically change during tumour progression from primary through to secondary metastasis and is inextricably linked to the inflammatory environment [249]. For example, many solid tumours (e.g., pancreatic, colon) are largely devoid of CTLs at the core of the tumour owing to the presence of TAMs, which secrete cytokines (interleukin-10 (IL10), TGFβ) that inhibit DC maturation and dampen effector T cell activity and infiltration [249]. As the tumour progresses, the number of immunosuppressive M2-like TAMs increases, which in turn promotes tumour progression by secreting EGF, VEGF, and MMPs; these factors promote cancer cell proliferation, angiogenesis, and invasion and a pre-metastatic niche, respectively. The authors would like to refer the reader to the following review articles that summarise some of the key properties of tumour infiltrating immune cells and the mechanisms behind immunosurveillance [249,250,251].

Of relevance to the current article, Ca^2+^ signalling is critical for almost every aspect of immune cell function, and most of these cells are particularly dependent on CRAC channel function [252,253,254,255]. For example, Ca^2+^ entry through CRAC channels in CTLs and NK cells is critical for their cytolytic immune cell functions [256]. Following formation of the immune synapse between the target cancer cell and the immune cell, antigen recognition and T cell receptor signaling trigger a CRAC-dependent increase in intracellular Ca^2+^ that stimulates lytic granule release proximal to the target cell [257]. Therefore, in addition to their opposing roles in proliferation/migration/invasion and apoptosis in cancer cells, CRAC channels have a further additional role in regulating immune surveillance and tumour elimination by immune cells.

Therefore, the critical role played by CRAC channels in immune cell function brings into question the potential benefits of CRAC/Orai channel inhibitors in reducing tumour growth, as proposed by numerous groups in the field [92,134,135,210,227,258,259]. This is because the putative therapeutic effects of CRAC/Orai blockers on cancer cells might be counteracted by a concomitant inhibitory effect on tumour-associated immune cells; CRAC/Orai blockade might be expected to reduce CTL and NK cell activity, thereby compromising the anti-tumour immune response. However, a recent study showed that CTLs and NK cells have a bell-shaped Ca^2+^-dependence with respect to cancer cell killing and lytic granule release [260]. In particular, CTLs have a very low optimum cytosolic Ca^2+^ (122–334 nM) and external Ca^2+^ (23–625 μM) dependency for efficient cancer cell elimination, the latter being well below the typical blood plasma Ca^2+^ concentration. While this raises questions as to the physiological relevance of CRAC/Orai channels in this response, it also suggests that partial inhibition of CRAC/Orai-linked Ca^2+^ signalling might actually be beneficial by shifting the Ca^2+^-dependency towards an optimum lytic granule release, and thus facilitate a more efficient cancer cell killing response by CTLs. Clearly, further research is required to fully characterize the effects of CRAC/Orai blockade on the immune response within tumours in vivo.

Paradoxically, some elements of the tumour-associated immune response can also facilitate tumour progression rather than combat it. This phenomenon could be at least in part attributable to changes in Ca^2+^ signalling dynamics, either within cancer cells or within the immune cells. For example, cytokines released by immune cells can induce changes in Ca^2+^ signalling within cancer cells, leading to migratory and invasive behaviour. Release of the inflammatory regulator C-C motif chemokine 18 [261] by TAMs stimulates Ca^2+^ signalling and facilitates migration of MDA-MB-231 breast cancer cells [262]. In this study, CCL18 release from TAMs led to activation of their putative cell surface receptor, phosphatidylinositol transfer protein membrane-associated 3 (PITPNM3; otherwise known as PYK2 N-terminal domain-interacting receptor 1, Nir1), which in turn phosphorylated PLCγ1 and PKCζ, activated IP3 kinase B1 (IP3KB1), and induced Ca^2+^ signalling. Importantly, CCL18 expression correlated with invasive behaviour and poor survival in patients with breast cancer. Similarly, release of the inflammatory chemokine C-C motif chemokine 5 (CCL-5) from TAMs induced migration of glioma cells in a process dependent on increases in cytosolic [Ca^2+^] and phosphorylation of CaMKII [263]. Indeed, CCL-5 has previously been implicated in invasive behaviour and metastasis in a range of tumours, including prostate, bone, and breast cancers [264].

On the other hand, evidence suggests that tumour cells can ‘hijack’ Ca^2+^ signalling within immune cells to enable evasion from the anti-tumour immune response. Prostaglandin E2 produced by MCF7 breast cancer cells stimulated upregulation of SERCA3 expression in CD4+ T cells, leading to ER stress and CD4+ T cell apoptosis [265]. Interestingly, the dihydropyrimidone derivative nifetepimine induced downregulation of SERCA3 expression, thereby protecting CD4+ T cells from tumour cell-induced cytotoxicity.

It is also important to note that the tumour microenvironment is notoriously highly acidic, owing to the highly glycolytic phenotype and increased lactic acid efflux exhibited by cancer cells [266]. This acidic microenvironment not only facilitates matrix remodelling, and thus migration/invasion [267], but also has a profound inhibitory effect on immune surveillance [251]. Specifically, tumour acidity inhibits the cancer cell killing properties of CTLs, NK cells, and antigen-presenting DCs, while simultaneously promoting the immunosuppressive properties of Tregs, myeloid cells, and TAMs [251]. This is particularly relevant because almost every Ca^2+^ channel/transporter is exquisitely sensitive to pH, especially CRAC channels [268,269], and the acid-sensing Ca^2+^-permeable ion channels (ASICs) have important roles in both cancer cells [270,271,272,273] and immune cells [274,275,276]. Moreover, the acidic tumour microenvironment would be expected to impact Ca^2+^-signalling in both cancer cells [232] and immune cells [277,278] by promoting PMCA activity. This is because ATP-driven PMCAs are Ca^2+^/H^+^ exchangers, and thus their Ca^2+^ efflux activity is facilitated by extracellular acidification and inhibited by extracellular alkalinisation [279,280]. Therefore, in addition to providing a privileged ATP supply to PMCAs, the highly glycolytic cancer cell phenotype would provide an abundance of extracellular H^+^ ions with which to accentuate PMCA activity, potentially enhancing apoptosis resistance in cancer cells or altering immune cell function. This means that any novel and specific PMCA inhibitors may be especially effective as a combination therapy with drugs that target aberrant tumour metabolism or tumour acidification. These include glycolytic inhibitors [244], lactic acid/monocarboxylate transporter (MCT) inhibitors [281], lactate dehydrogenase (LDH) inhibitors [282,283], or hypoxia inducible factor (HIF1α) inhibitors [284]. In addition, these drugs may also have a synergistic effect when combined with other novel immunotherapies such as immune checkpoint inhibitors [285].

While we currently have only a limited understanding of how immune response-related Ca^2+^ signalling is altered in cancer, these studies suggest that Ca^2+^ signalling modulators might find utility in ameliorating the dampened immune response observed in many tumours; at present, this is a burgeoning field that warrants further investigation.

## 8. Conclusions

It is clear that cytosolic Ca^2+^ signalling is central to the major hallmark processes of the cancer phenotype, including limitless replicative capacity, uncontrolled cell proliferation, resistance to apoptosis, tissue invasion, and angiogenesis, as well as immune surveillance [1]. Given that many of the mainstay anti-cancer drugs remain largely ineffective for many cancers, emerging scientific evidence points to the Ca^2+^ signalling machinery as a rich tapestry of novel putative therapeutic targets. Leading candidates include TRP channels, ARC channels (Orai3 and plasma membrane STIM1), plasma membrane SPCA2, and SOCE channels (ER STIM1 and Orai1). However, the ubiquitous nature of many Ca^2+^ channels and transporters means that targeting them with novel therapeutics may produce unacceptable adverse effects. This is especially true for SOCE, in which pharmacological blockade may successfully inhibit cell proliferation, migration, and invasion, but will potentially also lead to apoptosis resistance and facilitate immune evasion, while inhibiting critical functions in normal cells. Therefore, the ideal strategy is to target Ca^2+^ channels, or regulatory proteins or processes, that are either uniquely expressed or their expression results in an entirely new function in cancer cells. The latter seems to be certainly true for the plasma membrane SPCA2-mediated regulation of Orai1, which appears to be a unique property of breast cancer cells. Likewise, targeting the glycolytic ATP fuelling of the PMCA may also represent an effective therapeutic strategy for selectively targeting cancer cells. Nevertheless, many of the studies described in this review represent early stage basic science research, and thus it remains to be determined whether targeting these candidate proteins will be therapeutically effective in clinical studies of the human disease.

## Figures and Tables

**Table 1:** Altered expression of components of the Ca^2+^ signalling machinery during cancer. An increase or decrease in expression is indicated by the appropriate arrow. Each Ca^2+^ transporter is sub-categorised into store-operated Ca^2+^ channel (SOCE)/non-SOCE, transient receptor potential (TRP) channels, Ca^2+^ ATPases, and Ca^2+^ release channels. STIM, stromal interacting molecule; TRPV, TRP vanilloid; TRPM, TRP melastatin.

Ca^2+^ Transporter	Expression	Cancer Type	Reference
***SOCE/non-SOCE***			
Orai1	**↑**	Breast	[121,122,123]
	**↑**	Kidney	[124,125]
	**↑**	Prostate	[126]
	↓	Prostate	[127]
	**↑**	Oesophageal	[128]
Orai3	**↑**	Breast	[123,129,130,131]
	**↑**	Prostate	[126]
	↓	Prostate	[132]
	**↑**	Lung	[133]
STIM1	**↑**	Cervical	[134]
	**↑**	Breast	[122,135]
STIM2	↓	Breast	[122]
***TRP channels***	**↑**		
TRPC1	**↑**	Breast	[136]
TRPC2	**↑**	Breast	[137]
	**↑**	Ovarian	[138]
TRPC6	**↑**	Breast	[136,137]
	**↑**	Glioma	[139]
	**↑**	Liver	[140]
	**↑**	Oesophageal	[141]
TRPM7	**↑**	Pancreatic	[142]
	**↑**	Breast	[136]
	**↑**	Nasopharyngeal	[143]
TRPM8	**↑**	Pancreatic	[144]
	**↑**	Prostate	[145,146]
	**↑**	Breast	[136,146]
	**↑**	Melanoma	[146]
	**↑**	Colorectal	[146]
	**↑**	Lung	[146]
	**↑**	Glioblastoma	[147,148]
TRPV1	**↑**	Liver	[149]
	↓	Bladder	[150]
	**↑**	Prostate	[151]
TRPV2	**↑**	Prostate	[152,153]
TRPV6	**↑**	Breast	[136,154,155,156]
	**↑**	Prostate	[155,156]
	**↑**	Thyroid	[156]
	**↑**	Colon	[156]
	**↑**	Ovarian	[156]
***Ca^2+^ ATPase***			
PMCA2	**↑**	Breast	[157,158]
PMCA4	↓	Colon	[159]
SERCA2	↓	Oral	[160]
SERCA3	↓	Colon	[161]
	↓	Breast	[162]
***Ca^2+^ release channels***			
IP_3_R-1	↓	Glioblastoma	[163]
IP_3_R-2	**↑**		
IP_3_R-3	**↑**	Glioblastoma	[163]
	**↑**	Colon	[164]
